# Ring Opening upon Valence Shell Excitation in β-Butyrolactone: Experimental and Theoretical Methods

**DOI:** 10.3390/molecules30153137

**Published:** 2025-07-26

**Authors:** Pedro A. S. Randi, Márcio H. F. Bettega, Nykola C. Jones, Søren V. Hoffmann, Małgorzata A. Śmiałek, Paulo Limão-Vieira

**Affiliations:** 1 Departamento de Física, Universidade Federal do Paraná, Caixa Postal 19044, Curitiba 81531-980, PR, Brazil; pasr@fisica.ufpr.br; 2 ISA, Department of Physics and Astronomy, Aarhus University, Ny Munkegade 120, DK-8000 Aarhus, Denmark; nykj@phys.au.dk (N.C.J.); vronning@phys.au.dk (S.V.H.); 3 Faculty of Mechanical Engineering and Ship Technology, Institute of Naval Architecture, Gdansk University of Technology, Narutowicza 11/12, 80-233 Gdansk, Poland; 4 Department of Physics, Sophia University, Tokyo 102-8554, Japan; 5 Atomic and Molecular Collisions Laboratory, CEFITEC, Department of Physics, NOVA School of Science and Technology, Universidade NOVA de Lisboa, 2829-516 Caparica, Portugal

**Keywords:** β-butyrolactone, VUV, cross-sections, ab initio calculations, ring breaking

## Abstract

The valence-shell electronic state spectroscopy of β-butyrolactone (CH_3_CHCH_2_CO_2_) is comprehensively investigated by employing experimental and theoretical methods. We report a novel vacuum ultraviolet (VUV) absorption spectrum in the photon wavelength range from 115 to 320 nm (3.9–10.8 eV), together with ab initio quantum chemical calculations at the time-dependent density functional (TD-DFT) level of theory. The dominant electronic excitations are assigned to mixed valence-Rydberg and Rydberg transitions. The fine structure in the CH_3_CHCH_2_CO_2_ photoabsorption spectrum has been assigned to C=O stretching, $v_{7}^{\prime }\left(a\right)$, CH_2_ wagging, $v_{14}^{\prime }\left(a\right)$, C–O stretching, $v_{22}^{\prime }\left(a\right)$, and C=O bending, $v_{26}^{\prime }\left(a\right)$ modes. Photolysis lifetimes in the Earth’s atmosphere from 0 km up to 50 km altitude have been estimated, showing to be a non-relevant sink mechanism compared to reactions with the ^•^OH radical. The nuclear dynamics along the C=O and C–C–C coordinates have been investigated at the TD-DFT level of theory, where, upon electronic excitation, the potential energy curves show important carbonyl bond breaking and ring opening, respectively. Within such an intricate molecular landscape, the higher-lying excited electronic states may keep their original Rydberg character or may undergo Rydberg-to-valence conversion, with vibronic coupling as an important mechanism contributing to the spectrum.

## 1. Introduction

Beta-butyrolactone (β-butyrolactone), CH_3_CHCH_2_CO_2_, also known as 4-methyl-2-oxetanone, is a racemic mixture of (R)- and (S)-enantiomers and considered a potential carcinogenic chemical compound [1]. It has been used commercially in the plastic industry, as a solvent and as a sterilant due to its chemical reactivity [2]. β-butyrolactone is also relevant within the chemistry of the Earth’s troposphere, undergoing tropospheric oxidation through reactions with ^•^OH radicals [3].

Recently, we have investigated the electronic and molecular properties of β-propiolactone, which is more reactive with nucleic acids than β-butyrolactone [1,2], with novel absolute cross-section values from vacuum ultraviolet photoabsorption in the photon energy range 4.6–10.8 eV [4]. Ring strain instability was carefully assessed with the aid of quantum chemical calculations showing the most favourable route along the C–O ring bond excision [4].

Molecular compounds from either anthropogenic or natural emissions can play a relevant role in the Earth’s atmosphere’s local chemistry and physics. Since establishing an international partnership to investigate the electronic state spectroscopy of polyatomic molecules with atmospheric relevance (see e.g., [5,6]), our main interest has been to provide the most accurate absolute cross-section values in the vacuum ultraviolet (VUV). These, in combination with the solar actinic flux and the quantum yields for dissociation (see Section 3.6), have on several occasions allowed the estimation of atmospheric lifetimes from 0 up to 50 km altitude [6,7,8,9,10,11,12,13,14,15,16] and references therein. Other sink mechanisms have also been evaluated, in particular from the available data in the literature on the chemical reactivity with the major radicals present in the Earth’s atmosphere, e.g., ^•^OH and Cl [17]. The present β-butyrolactone absorption spectrum in the photon energy region from 3.9 eV (320 nm) to 10.8 eV (115 nm) will be used to estimate the molecular compound’s lifetime in the terrestrial atmosphere.

The detailed analysis and assignment of the vibronic features in the VUV spectrum are supported by available complementary spectroscopic data in the literature. Relevant to the assignments are experimental infrared and Raman spectroscopies [18]. Theoretical studies include calculations of the barrier to internal rotation [18,19], equilibrium geometries [19,20], and ab initio calculations of vibrational absorption [21,22]. Finally, the rate of reaction with ^•^OH radicals has been reported by Barnes et al. [3].

The structure of this paper includes Section 2 dealing with the electronic and molecular structure of β-butyrolactone, whereas Section 3 presents the results and discussion of the main electronic excitations and the assignment of the fine structure. Section 4 gives a short description of the experimental and theoretical methodologies. We conclude in Section 5 with the overall analysis on the valence-shell excitation of CH_3_CHCH_2_CO_2_.

## 2. Structure and Properties of β-Butyrolactone

The β-butyrolactone neutral ground state and cationic ground state geometries (bond lengths and bond angles) obtained at the DFT/CAM-B3LYP/aug-cc-pVTZ level of theory in the *C*_1_ symmetry group are shown in Figures S1 and S2. The calculated outermost valence orbital electronic configuration of X~A1 ground state is … (18a)^2^ (19a)^2^ (20a)^2^ (21a)^2^ (22a)^2^ (23a)^2^. For the complete configuration, see the Supplementary Material.

**Figure 1:**
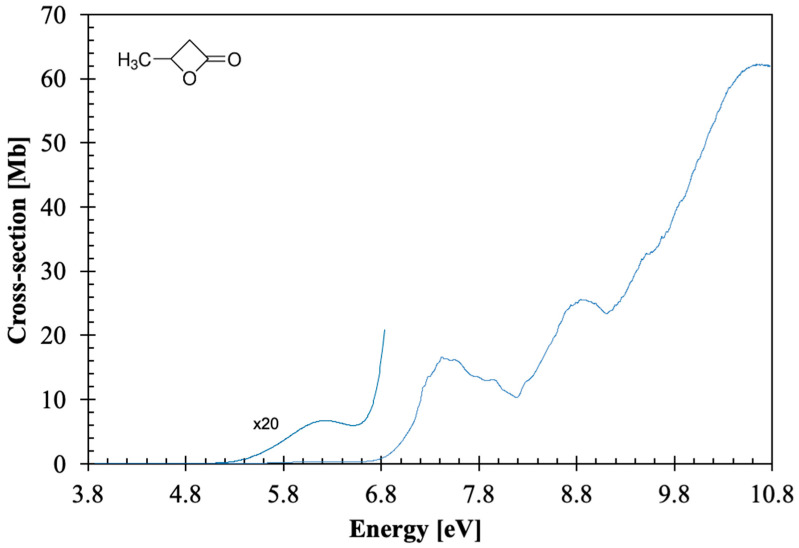
The photoabsorption spectrum of β-butyrolactone, CH_3_CHCH_2_CO_2_, in the 3.8–10.8 eV photon energy range.

**Figure 2:**
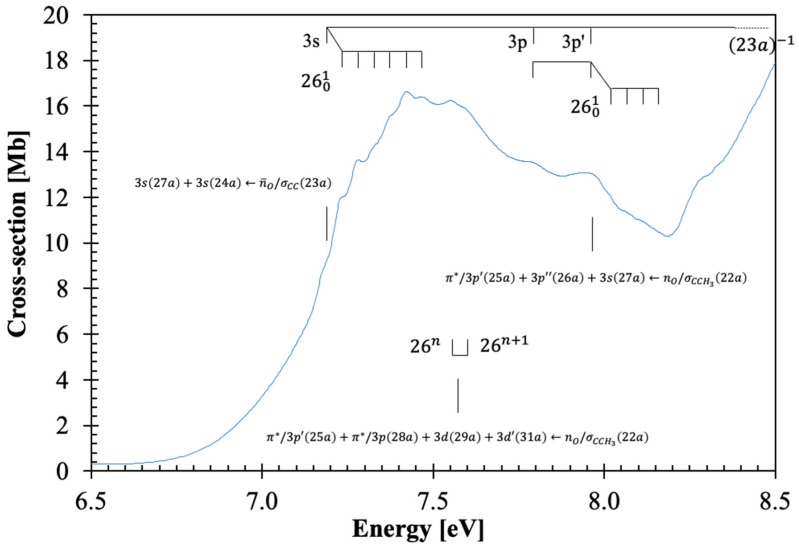
Enlarged section of the photoabsorption spectrum of β-butyrolactone, CH_3_CHCH_2_CO_2_, in the 6.5–8.5 eV photon energy range.

**Figure 3:**
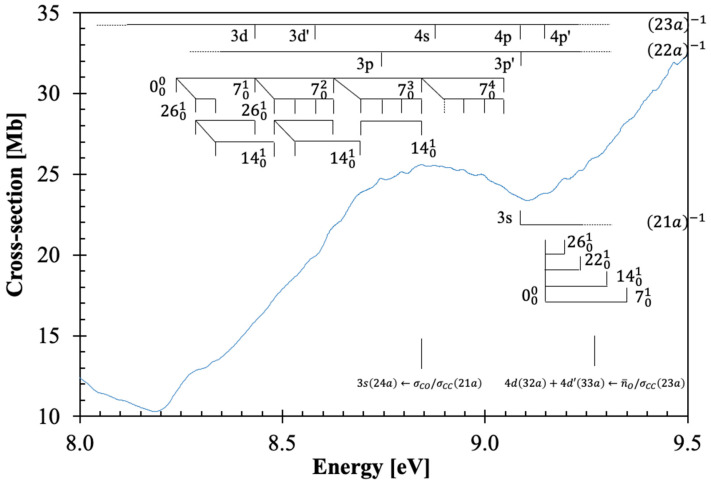
Enlarged section of the photoabsorption spectrum of β-butyrolactone, CH_3_CHCH_2_CO_2_, in the 8.0–9.5 eV photon energy range. The vertical dashed lines mean tentative assignments of less resolved features. See text for details.

**Figure 4:**
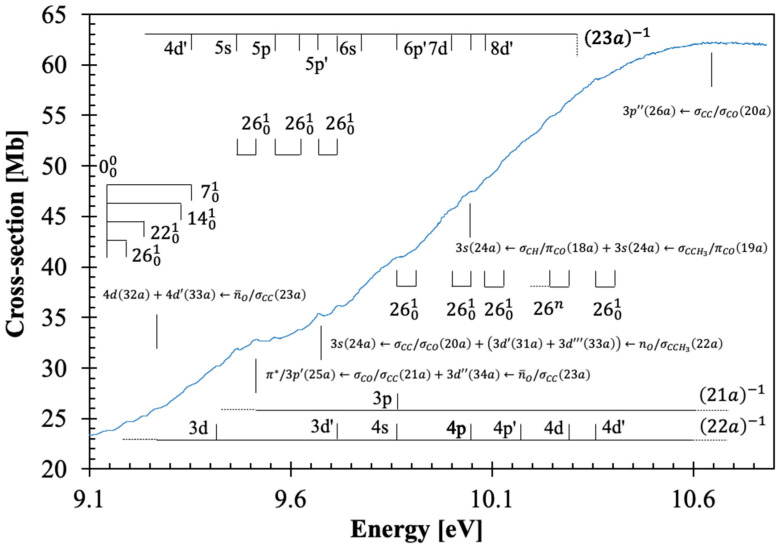
Enlarged section of the photoabsorption spectrum of β-butyrolactone, CH_3_CHCH_2_CO_2_, in the 9.1–10.8 eV photon energy range.

The calculated vertical excitation energies and oscillator strengths of β-butyrolactone are shown in Table S1 (TD-DFT/CAM-B3LYP/aug-cc-pVTZ), while the assignment of the major electronic excitations in the photoabsorption spectrum in Figure 1, Figure 2, Figure 3 and Figure 4 can be found in Table 1. Moreover, Table S2 contains the eight lowest-lying electronic excitations obtained at the EOM-CCSD/aug-cc-pVDZ level whereas Table 2, Table 3 and Table 4 contain the proposed vibrational progressions.

The electronic transitions from the ground state involve (Figure S3) the (HOMO), 23a, oxygen lone-pair in-plane/C–C, ${\bar{n}}_{O}/\sigma_{CC}$, the (HOMO-1), 22a, the oxygen lone-pair out-of-plane/C–CH_3_, $n_{O}/\sigma_{CCH_{3}}$, the (HOMO-2) and (HOMO-3), 21a and 20a, $\sigma_{CO}/\sigma_{CC}$, the (HOMO-4), 19a, $\sigma_{CCH_{3}}/\pi_{CO}$ and the (HOMO-5), 18a, $\sigma_{CH}/\pi_{CO}$ to mixed valence-Rydberg and Rydberg character orbitals (Table 1). Harmonic frequencies at the DFT level with the CAM-B3LYP/aug-cc-pVTZ functional and basis set for the neutral electronic (Table S3) and cation electronic ground state (Table S4) have been calculated; the former was also compared with the infrared spectroscopy data of Durig et al. [18]. The vibrational excitation discernible in the spectrum from 7.0 up to 10.8 eV (Figure 2, Figure 3 and Figure 4) is mostly related to the Rydberg character of the electronic transitions and has been assigned from the information in Table S4 to 0.198 eV (1594 cm^−1^) for C=O stretching, $v_{7}^{\prime }\left(a\right)$, 0.153 eV (1232 cm^−1^) for CH_2_ wagging, $v_{14}^{\prime }\left(a\right)$, 0.096 eV (775 cm^−1^) for C–O stretching, $v_{22}^{\prime }\left(a\right)$ and 0.052 eV (416 cm^−1^) for C=O bending, $v_{26}^{\prime }\left(a\right)$. Note that in the cation, C=O bending mode becomes $v_{27}^{\prime }\left(a\right)$; however, we have kept the mode numbering according to the neutral ground state assignment and consistent with Durig et al.’s [18].

The tentative assignment of the different Rydberg series in Table 5 has been performed from the quantum defects obtained with the calculated ionization adiabatic/vertical energies at the TD-DFT/CAM-B3LYP/aug-cc-pVTZ level of theory (IE_ad_/IE_v_), (IE_1_)_ad_ = 10.31 eV (23a)^−1^, (IE_2_)_v_ = 11.29 eV (22a)^−1^, and (IE_3_)_v_ = 12.47 eV (21a)^−1^ (see Section 4), since no experimental data is available in the literature.

## 3. Discussion

The β-butyrolactone high-resolution absorption spectrum, in the photon energy range from 3.9 to 10.8 eV (320–115 nm), is shown in Figure 1, while extended sections showing the electronic transitions, vibrational structure, and Rydberg assignments are shown in Figure 2, Figure 3 and Figure 4. The assignment of the electronic excitations from the neutral ground state to mixed valence-Rydberg and Rydberg states (Table 1) has been performed with the aid of TD-DFT/CAM-B3LYP/aug-cc-pVTZ calculations (Section 4), whereas complementary EOM-CCSD/aug-cc-pVDZ calculations have been performed up to 8.7 eV (Table S2). These also provide vertical excitation energies and oscillator strengths, are compared with the experimental data and the related absolute cross-section values. The results from the calculations for all electronic transitions in Table 1 and Table S2 show a good level of agreement with the experimental data to within 7%. The photoabsorption spectrum above 7 eV is mainly due to Rydberg excitations, so the fine structure (Figure 2, Figure 3 and Figure 4) has been assigned (Table 2, Table 3 and Table 4) from the harmonic frequencies calculated at the DFT level with the CAM-B3LYP/aug-cc-pVTZ functional and basis set for β-butyrolactone ionic electronic ground state (Table S4).

**Table 2:** Proposed vibrational assignments of the β-butyrolactone, CH_3_CHCH_2_CO_2_, absorption band in the photon energy range 6.5–8.5 eV ^a^. Energies in eV. See text for details.

Assignment	Energy	$\Delta E\left(\nu_{7}^{\prime }\right)$	$\Delta E\left(\nu_{14}^{\prime }\right)$	$\Delta E\left(\nu_{22}^{\prime }\right)$	$\Delta E\left(\nu_{26}^{\prime }\right)$
3s27a+3s24a←n¯O/σCC23a, 3A1←X~A1					
$0_{0}^{0}/3s{\left(23a\right)}^{-1}$	7.19(0) (s)	-	-	-	-
$26_{0}^{1}$	7.24(0) (s)	-	-	-	0.050
$26_{0}^{2}/22_{0}^{1}$	7.285	-	-	0.095	0.045
$26_{0}^{3}/14_{0}^{1}$	7.33(4) (s,w)	-	0.144	-	0.049
$26_{0}^{4}/22_{0}^{2}/7_{0}^{1}$	7.37(8) (s)	0.188	-	0.093	0.053
$26_{0}^{5}$	7.424	-	-	-	0.046
$26_{0}^{6}/22_{0}^{3}/14_{0}^{2}$	7.47(1) (w)	-	0.137	0.093	0.047
π∗/3p′25a+π∗/3p28a+3d29a+3d′31a←nO/σCCH322a, 6A1←X~A1					
$26^{n}$	7.55(5) (b)	-	-	-	-
$26^{n+1}$	7.59(7) (s)	-	-	-	0.042
π∗/3p′25a+3p″26a+3s27a←nO/σCCH322a, 9A1←X~A1					
$0_{0}^{0}/3p{\left(23a\right)}^{-1}$	7.78(6) (b)	-	-	-	-
$7_{0}^{1}/3p^{\prime }{\left(23a\right)}^{-1}$	7.94(5) (b)	0.159	-	-	-
$7_{0}^{1}26_{0}^{1}$	8.01(2) (s,b)	-	-	-	0.067
$7_{0}^{1}26_{0}^{2}/7_{0}^{1}22_{0}^{1}$	8.06(4) (s)	-	-	0.106	0.052
$7_{0}^{1}26_{0}^{3}/7_{0}^{1}14_{0}^{1}$	8.11(2) (s)	-	0.154	-	0.048
$7_{0}^{1}26_{0}^{4}/7_{0}^{1}22_{0}^{2}$	8.15(7) (s,w)	-	-	0.093	0.045
	$\bar{\Delta E}$	0.174	0.145	0.096	0.050

^a^ (s) shoulder structure; (w) weak feature; (b) broad feature (the last decimal of the energy value is given in brackets for these less-resolved features).

**Table 3:** Proposed vibrational assignments of the β-butyrolactone, CH_3_CHCH_2_CO_2_, absorption band in the photon energy range 8.0–9.5 eV ^a^. Energies in eV. See text for details.

Assignment	Energy	$\Delta E\left(\nu_{7}^{\prime }\right)$	$\Delta E\left(\nu_{14}^{\prime }\right)$	$\Delta E\left(\nu_{22}^{\prime }\right)$	$\Delta E\left(\nu_{26}^{\prime }\right)$
3s24a←σCO/σCC21a, 14A1←X~A1					
$0_{0}^{0}$	8.23(8) (s,w)	-	-	-	-
$26_{0}^{1}$	8.28(8) (s)	-	-	-	0.050
$26_{0}^{2}$	8.33(5) (s)	-	-	-	0.047
$7_{0}^{1}/26_{0}^{1}14_{0}^{1}/3d{\left(23a\right)}^{-1}$	8.43(1) (s,w)	0.193	0.143	-	-
$7_{0}^{1}26_{0}^{1}/26_{0}^{1}3d{\left(23a\right)}^{-1}/26_{0}^{2}14_{0}^{1}$	8.48(1) (s,w)	-	0.146	-	0.050
$7_{0}^{1}26_{0}^{2}/26_{0}^{2}3d{\left(23a\right)}^{-1}$	8.53(3) (s,w)	-	-	-	0.052
$7_{0}^{1}26_{0}^{3}/26_{0}^{3}3d{\left(23a\right)}^{-1}/3d{\prime \left(23a\right)}^{-1}$	8.58(0) (s)	-	-	-	0.047
$7_{0}^{2}/7_{0}^{1}26_{0}^{4}/26_{0}^{4}3d{\left(23a\right)}^{-1}/7_{0}^{1}26_{0}^{1}14_{0}^{1}$	8.62(9) (s)	0.198	0.148	-	0.049
$7_{0}^{2}26_{0}^{1}$	8.69(2) (s,b)	-	0.159	-	0.063
$7_{0}^{2}26_{0}^{2}/3p{\left(22a\right)}^{-1}$	8.74(4) (b)	-	-	-	0.052
$7_{0}^{2}26_{0}^{3}$	8.795	-	-	-	0.051
$7_{0}^{3}/7_{0}^{2}26_{0}^{4}/7_{0}^{2}26_{0}^{1}14_{0}^{1}$	8.84(2) (x)	0.213	0.150	-	0.047
$7_{0}^{3}26_{0}^{1}$	8.89(7) (?)	-	-	-	0.055
$7_{0}^{3}26_{0}^{2}$	8.94(6) (b)	-	-	-	0.049
$7_{0}^{3}26_{0}^{3}$	8.99(4) (b)	-	-	-	0.048
$7_{0}^{4}/7_{0}^{3}26_{0}^{4}$	9.04(1) (s,w)	0.199	-	-	0.047
4d32a+4d′33a←n¯O/σCC23a, 17A1←X~A1					
$0_{0}^{0}/4p^{\prime }{\left(23a\right)}^{-1}$	9.14(6) (b)	-	-	-	-
$26_{0}^{1}$	9.195	-	-	-	0.049
$22_{0}^{1}$	9.23(5) (w)	-	-	0.089	-
(?)	9.26(9) (b)	-	-	-	-
$14_{0}^{1}$	9.30(1) (s,w)	-	0.155	-	-
$7_{0}^{1}/4d^{\prime }{\left(23a\right)}^{-1}$	9.35(0) (b,w)	0.204	-	-	-
$7_{0}^{1}22_{0}^{1}/4d^{\prime }{\left(23a\right)}^{-1}/3d{\left(22a\right)}^{-1}$	9.42(4) (s,b)	-	-	0.074	-
$7_{0}^{1}22_{0}^{1}26_{0}^{1}/4d^{\prime }{\left(23a\right)}^{-1}26_{0}^{1}/5s{\left(23a\right)}^{-1}$	9.467	-	-	-	0.043
	$\bar{\Delta E}$	0.201	0.150	0.082	0.050

^a^ (s) shoulder structure; (w) weak feature; (b) broad feature; (x) hardly visible; (?) unassigned feature (the last decimal of the energy value is given in brackets for these less-resolved features).

**Table 4:** Proposed vibrational assignments of the β-butyrolactone, CH_3_CHCH_2_CO_2_, absorption band in the photon energy range 9.4–10.8 eV ^a^. Energies in eV. See text for details.

Assignment	Energy	$\Delta E\left(\nu_{26}^{\prime }\right)$
$5s{\left(23a\right)}^{-1}$	9.467	-
$26_{0}^{1}$	9.51(7) (w)	0.050
$26_{0}^{2}/5p{\left(23a\right)}^{-1}$	9.56(4) (w)	0.047
$5p^{\prime }{\left(23a\right)}^{-1}$	9.62(8) (w)	-
$26_{0}^{1}/5d{\left(23a\right)}^{-1}/3d^{\prime }{\left(22a\right)}^{-1}$	9.673	0.045
$26_{0}^{2}/5d^{\prime }{\left(23a\right)}^{-1}/26_{0}^{1}5d{\left(23a\right)}^{-1}/26_{0}^{1}3d^{\prime }{\left(22a\right)}^{-1}$	9.723	0.050
$6p^{\prime }{\left(23a\right)}^{-1}/4s{\left(22a\right)}^{-1}/3p{\left(21a\right)}^{-1}$	9.86(4) (b,w)	-
$26_{0}^{1}$	9.91(4) (s,w)	0.050
$7d{\left(23a\right)}^{-1}$	10.00(2) (s)	-
$26_{0}^{1}/8p{\left(23a\right)}^{-1}/4p{\left(22a\right)}^{-1}$	10.05(1) (b,w)	0.049
$8d^{\prime }{\left(23a\right)}^{-1}$	10.08(7) (s,w)	-
$26_{0}^{1}$	10.13(4) (s,w)	0.047
$26^{n}$	10.24(7) (s)	-
$26^{n+1}/4d{\left(22a\right)}^{-1}$	10.29(6) (b,w)	0.049
$4d^{\prime }{\left(22a\right)}^{-1}$	10.360	-
$26_{0}^{1}$	10.41(0) (w)	0.050
	$\bar{\Delta E}$	0.049

^a^ (b) broad feature; (s) shoulder structure; (w) weak feature (the last decimal of the energy value is given in brackets for these less-resolved features);.

A close comparison of the β-butyrolactone and β-propiolactone [4] absorption spectra shows the latter with more enhanced fine structure. This seems reasonable given the longer side chain in β-butyrolactone relative to β-propiolactone, which yields more degrees of freedom, and the contribution of these tends to blur the absorption spectrum. An identical behavior has been reported for fatty acids by increasing the side chain in the sequence propionic < butyric < valeric acids [23].

The Rydberg character of the absorption features has been tested from the quantum defects calculated for each series converging to the different ionic electronic states (23a)^−1^ X~A2, (22a)^−1^ A~A2 and (21a)^−1^ B~A2 (see Table 5 and Section 3.4). A general overview of the β-butyrolactone absorption spectrum above 7 eV shows the baseline constantly shifted as the photon energy increases. The valence nature of the electronic excitations in Table 1 are $\pi^{*}$ in character, which in principle do not yield dissociative states. Table S1 lists the complete set of electronic excitations, and a close inspection of the unoccupied MOs in Figure S3 shows the rather pronounced Rydberg character with less distinct $\sigma^{*}$ antibonding MOs. Thus, upon electronic excitation from the ground state to a $\pi^{*}$ MO, as long as the nuclear wave packet survives long enough along the reaction coordinate, the system can diabatically cross with such antibonding MOs that will lead to bond excision. These may be responsible for the background continuum in the photoabsorption cross-section profile. Of note, an identical behavior of the electronic transitions has recently been observed for β-propiolactone [4].

Another interesting aspect of β-butyrolactone spectroscopy is the lack of any rotational envelope structure for the lowest-lying excited state, which has been observed in other cyclic and non-cyclic molecules, e.g., [15,24,25,26,27,28]. However, this is not unexpected given the internal barrier for CH_3_ rotation of 3.3 ± 0.3 kcal/mol [18] and 3.590 kcal/mol [19].

The next section contains the assignment of the different absorption bands of β-butyrolactone in different photon energy ranges, where a complete description of the dominant electronic (and vibrational) excitations and their origins is reported, together with absolute cross-section values and the expected atmospheric sink mechanisms.

### 3.1. The 3.8–6.5 eV Photon Energy Range

The calculated lowest-lying triplet state is obtained at 5.452 eV, assigned mainly to the πCO∗30a←n¯O/σCC23a, 1A3←X~A1 transition, at the TD-DFT/CAM-B3LYP/aug-cc-pVTZ level, and at 5.816 eV through an EOM-CCSD/aug-cc-pVDZ level of calculation. The nature of the electronic transitions in a photoabsorption spectrum are typically due to optically allowed transitions, while those that are optically forbidden transitions are not usually discernible. The rather low cross-section of the band (<0.01 Mb) can also be related in some way to a contribution of this kind. Recently, we have reported an identical behavior in the lowest absorption band of formic acid [29]. A literature survey reveals no electron energy loss spectroscopy measurements to confirm the presence of this state.

The lowest-lying valence singlet excitation is assigned to an electron promotion from the (HOMO), 23a, ${\bar{n}}_{O}/\sigma_{CC}$, orbital to a mixed valence-Rydberg and Rydberg molecular orbital π∗/3p28a+3d29a+3d′31a←n¯O/σCC23a, 2A1←X~A1 (Table 1 and Table S2) peaking at 6.212 eV and with a cross-section value of 0.39 Mb (Figure 1). The calculated vertical excitation energy of 6.010 eV, with an oscillator strength *f*_L_ = 0.00047, is ~3% lower than the experimental value. The absorption band is structureless and of low intensity, suggesting that it may be mainly due to the $\pi^{*}$ valence character, because the first member of the Rydberg series converging to the lowest ionization energy assigned to 3s is at 7.19(0) eV (Section 3.5). A similar behaviour was also observed for the first absorption band of β-propiolactone [4].

### 3.2. The 6.5–8.2 eV Photon Energy Range

This energy range comprises three electronic transitions as shown in Figure 2. The first excitation is assigned to an electron promotion from the (HOMO), 23a, ${\bar{n}}_{O}/\sigma_{CC}$, to the LUMO and LUMO+3 Rydberg molecular orbitals (Table 1 and Table S2 and Section 3.5), 3s27a+3s24a←n¯O/σCC23a, 3A1←X~A1, with a cross-section value of 16.63 Mb at 7.424 eV (Figure 2). The calculated vertical excitation energy of 7.370 eV and an oscillator strength of *f*_L_ = 0.02663, is <1% lower than the experimental value. We tentatively assign the $0_{0}^{0}$ origin at 7.19(0) eV (Table 2), showing up to six quanta of the C=O bending $v_{26}^{\prime }\left(a\right)$ mode and/or up to three quanta of CH_2_ wagging, $v_{14}^{\prime }\left(a\right)$ mode. The features can also be assigned to combinations of C=O stretching, $v_{7}^{\prime }\left(a\right)$, and C–O stretching, $v_{22}^{\prime }\left(a\right)$ modes. The average spacings of $v_{14}^{\prime }\left(a\right)$, $v_{22}^{\prime }\left(a\right)$ and $v_{26}^{\prime }\left(a\right)$ are 0.141 eV (1137 cm^−1^), 0.094 eV (758 cm^−1^), and 0.048 eV (387 cm^−1^). The first member of the *ns* Rydberg series converging to the (23a)^−1^ X~A2 ionic electronic ground state at 7.19(0) eV is discussed in Section 3.5.

The second absorption band, tentatively centered at 7.55(5) eV with a maximum cross-section of 16.21 Mb, is assigned to a mixed valence-Rydberg and Rydberg character π∗/3p′25a+π∗/3p28a+3d29a+3d′31a←nO/σCCH322a, 6A1←X~A1 with an oscillator strength of 0.06959 (Table 1). Due to the proximity with the previous band, we are not able to assign the $0_{0}^{0}$ origin (Figure 2), and so the broad feature, which is due to C=O bending, $v_{26}^{\prime }\left(a\right)$ mode, is assigned as $26^{n}$ (Table 2).

From the theoretical calculations in Table 1, the next absorption band in this photon energy region is assigned to a mixed valence-Rydberg and Rydberg excitation, π∗/3p′25a+3p″26a+3s27a←nO/σCCH322a, 9A1←X~A1. The band peaks at 7.94(5) eV with a cross-section value of 13.09 Mb, with a calculated vertical excitation energy of 8.585 eV and an oscillator strength of 0.03097. The band is mainly due to Rydberg contributions (Section 3.5) and exhibits weak vibrational fine structure of C=O bending, $v_{26}^{\prime }\left(a\right)$ mode, and combinations with CH_2_ wagging, $v_{14}^{\prime }\left(a\right)$ and C–O stretching, $v_{22}^{\prime }\left(a\right)$ modes, with mean energy values of 0.053, 0.154, and 0.100 eV, respectively (Table 2).

### 3.3. The 8.5–9.4 eV Photon Energy Range

This energy range includes two electronic excitations centered at 8.842 and 9.272 eV (Figure 3), which are assigned to Rydberg transitions (see Section 3.5), with cross-section values of 25.58 and 33.96 Mb (Table 1 and Figure 3). The character of the calculated electronic transitions is 3s24a←σCO/σCC21a, 14A1←X~A1 and 4d32a+4d′33a←n¯O/σCC23a, 17A1←X~A1. The $0_{0}^{0}$ origin bands are assigned at 8.23(8) and 9.14(6) eV and are accompanied by excitation of C=O stretching, $v_{7}^{\prime }\left(a\right)$, CH_2_ wagging, $v_{14}^{\prime }\left(a\right)$, C–O stretching, $v_{22}^{\prime }\left(a\right)$, and C=O bending, $v_{26}^{\prime }\left(a\right)$ modes, and combinations of these, with mean energy values of 0.201, 0.150, 0.082, and 0.050 eV, respectively (Table 3). The vertical dashed line in Figure 3 indicates a tentative assignment of the less resolved feature.

### 3.4. The 9.4–10.8 eV Photon Energy Range

This energy range contains five electronic transitions assigned in Table 1 and with the majority of these as shown in Figure 4. These are mainly Rydberg (Section 3.5), with the first also showing a mixed valence-Rydberg character, followed by vibrational fine structure (Table 4).

The assigned electronic states peak at 9.515, 9.670, 9.87(1), 10.04(7), and 10.69(8) eV, with local cross-section values of 32.82, 35.40, 41.01, 47.47, and 62.27 Mb, and with calculated oscillator strengths of 0.02452, 0.03017, 0.03858, 0.04253, and 0.07819, respectively. These transitions are assigned to π∗/3p′25a←σCO/σCC21a+3d″34a←n¯O/σCC23a, 24A1←X~A1, 3s24a←σCC/σCO20a+3d′31a+3d‴33a←nO/σCCH322a, 28A1←X~A1, nf′35a+nf40a←n¯O/σCC23a, 31A1←X~A1, 3s24a←σCH/πCO18a+3s24a←σCCH3/πCO19a, 36A1←X~A1, and 3p″26a←σCC/σCO20a, 40A1←X~A1, and show fine structure superimposed on the Rydberg series (see Section 3.5). As reported in the spectroscopy of β-propiolactone [4], the majority of the Rydberg features above 9.3 eV appear quite broad and barely discernible in the high-energy region of the photoabsorption band. This is indicative of different members of the Rydberg series contributing to the absorption spectrum, the evidence of vibrational excitation, and, no less relevant the $\sigma^{*}$ antibonding characters (see Table S1).

### 3.5. Rydberg Transitions

The different Rydberg transitions above 7.0 eV have been assigned in the photoabsorption spectrum (Table 5), with their fine structure in Figure 2, Figure 3 and Figure 4 and Table 2, Table 3 and Table 4. The Rydberg series converging to the (23a)^–1^ X~A2, (22a)^–1^ A~A,2 and (21a)^–1^ B~A2 ionic electronic states of β-butyrolactone have been assigned according to their positions and the quantum defects obtained from the Rydberg formula: $E_{n}=IE-R/{\left(n-\delta \right)}^{2}$, with *IE* the ionization energy of a given MO, *n* is the principal quantum number of the Rydberg orbital of energy *E*_n_, *R* is the Rydberg constant (13.61 eV), and *δ* is the quantum defect resulting from the penetration of the Rydberg orbital into the core.

**Table 5:** Energy values (*E*_n_ in eV), quantum defects (*δ*), and assignments (asg.) of the Rydberg series converging to (23a)^−1^ X~A2, (22a)^−1^ A~A2, and (21a)^−1^ B~A2 of β-butyrolactone, CH_3_CHCH_2_CO_2_. See text for details.

*E*_n_	*δ*	Asg.	*E*_n_	*δ*	Asg.	*E*_n_	*δ*	Asg.	*E*_n_	*δ*	Asg.	*E*_n_	*δ*	Asg.
$({\mathrm{IE}}_{1}{)}_{\mathrm{ad}}=10.31\;\mathrm{eV}\;{\left(23a\right)}^{-1}$														
(*ns* ← *23a*)			(*np* ← *23a*)			(*np*′ ← *23a*)			(*nd* ← *23a*)			(*nd*′ ← *23a*)		
7.19(0) (s)	0.91	3s	7.78(6) (b)	0.68	3p	7.94(5) (b)	0.60	3p′	8.43(1) (s,w)	0.31	3d	8.58(0) (s)	0.20	3d′
8.87(8) (w)	0.92	4s	9.07(9) (s,w)	0.67	4p	9.14(6) (b)	0.58	4p′	-	-	4d	9.35(0) (b,w)	0.23	4d′
9.467	0.98	5s	9.56(4) (w)	0.73	5p	9.62(8) (w)	0.53	5p′	9.673	0.38	5d	9.723	0.18	5d′
9.78(0) (s,w)	0.93	6s	-	-	6p	9.86(4) (b,w)	0.48	6p′	-	-	6d	-	-	6d′
-	-	-	-	-	-	-	-	-	10.00(2) (s)	0.35	7d	-	-	7d′
-	-	-	10.05(1) (b,w)	0.75	8p	-	-	-	-	-	-	10.08(7) (s,w)	0.19	8d′
**(IE_2_)_v_ = 11.29 eV** ${\left(22a\right)}^{-1}$														
(*ns* ← *22a*)			(*np* ← *22a*)			(*np*′ ← *22a*)			(*nd* ← *22a*)			(*nd*′ ← *22a*)		
-	-	3s	8.74(4) (b)	0.69	3p	9.07(9) (s,w)	0.52	3p′	9.42(4) (s,b)	0.30	3d	9.673	0.10	3d′
9.86(4) (b,w)	0.91	4s	10.05(1) (b,w)	0.68	4p	10.17(1) (s,w)	0.51	4p′	10.29(6) (b,w)	0.30	4d	10.360	0.17	4d′
**(IE_3_)_v_ = 12.47 eV** ${\left(21a\right)}^{-1}$														
(*ns* ← *21a*)			(*np* ← *21a*)			-			-			-		
9.07(9) (s,w)	1.00	3s	9.86(4) (b,w)	0.71	3p	-	-	-	-	-	-	-	-	-

(s) shoulder structure; (w) weak feature; (b) broad structure (the last decimal of the energy value is given in brackets for these less-resolved features).

The lowest-lying Rydberg transition (*n* = 3) converging to the ionic electronic ground state is assigned to the $\left(3s\leftarrow 23a\right)$ excitation, with the first member at 7.19(0) eV having a quantum defect *δ* = 0.91. Higher-order Rydberg members of the ns series up to *n* = 6 are reported in Table 5. The first members of the two *np* $\left(np\leftarrow 23a\right)$ and $\left(np^{\prime }\leftarrow 23a\right)$ series have absorption features at 7.78(6) and 7.94(5) eV (*δ* = 0.68 and 0.60). The other two are *nd* $\left(nd\leftarrow 23a\right)$ and $\left(nd^{\prime }\leftarrow 23a\right)$ series with principal quantum numbers up to *n* = 8, where *n* = 3 have been assigned at 8.43(1) and 8.58(0) eV (*δ* = 0.31 and 0.20). The features at 9.07(9), 9.86(4) and 10.05(1) eV can also be assigned to ${3p^{\prime }\left(22a\right)}^{-1}/{3s\left(21a\right)}^{-1}$, ${4s\left(22a\right)}^{-1}/{3p\left(21a\right)}^{-1}$ and ${4p\left(22a\right)}^{-1}$. The feature at 9.673 eV is too intense for a 5d Rydberg member and is therefore assigned to ${3d^{\prime }\left(22a\right)}^{-1}$.

The Rydberg series converging to the ionic electronic first excited state IE_2_, (22a)^−1^, are listed in Table 5, and have been assigned to the $\left(ns,\;np,np^{\prime },nd,nd^{\prime }\leftarrow 22a\right)$ transitions. The members of these series for *n* = 3 (except for 3s) are associated with features at 8.74(4), 9.07(9), 9.42(4), and 9.673 eV with quantum defects *δ* = 0.69, *δ* = 0.52, *δ* = 0.30, and *δ* = 0.10, respectively (Table 5). Higher members of these series for *n* > 4 lie outside the energy range of the photoabsorption spectrum.

The Rydberg series converging to the ionic electronic second excited state IE_3_, ${\left(21a\right)}^{-1}$, are listed in Table 5 and have been assigned to the $\left(ns,\;np\leftarrow 21a\right)$ transitions. The first members of these series (*n* = 3) are associated with features at 9.07(9) eV (*δ* = 1.00) and 9.86(4) eV (*δ* = 0.71).

As discussed before, the dominant electronic excitations in the VUV photoabsorption spectrum have been assigned to Rydberg transitions with fine structure superimposed on these. To check the contribution of the main vibrational modes shown in Figure 2, Figure 3 and Figure 4 and listed in Table 2, Table 3 and Table 4, the neutral and cationic ground state geometries (Figures S1 and S2) have been obtained at the DFT/CAM-B3LYP/aug-cc-pVTZ level of theory. The optimized bond lengths in Å and bond angles in (°) are shown in Figures S1 and S2. A close inspection of the data between neutral and cation shows that upon ionization, shortening up to 6% is noted within the O1-C2 bond length, whereas an increase of 2 and 5% occurs for C2-O6 and O1-C3, and a 2% increase is observed for the mutual distance between C2-C4. Such changes are in assertion of the assignments proposed in Table 2, Table 3 and Table 4 and related to C=O stretching, $v_{7}^{\prime }\left(a\right)$, C-O stretching, $v_{22}^{\prime }\left(a\right)$ and C=O bending, $v_{26}^{\prime }\left(a\right)$. Moreover, an increase of 5% within the O1-C2-O6, an increase of 4% in O1-C2-O4, and a shortening of ≈ 3% in the O1-C3-H7 and C2-C4-H8 angles are consistent with the general ring (and molecular structure) deformation related to C=O stretching, $v_{7}^{\prime }\left(a\right)$, CH_2_ wagging, $v_{14}^{\prime }\left(a\right)$, C-O stretching, $v_{22}^{\prime }\left(a\right)$ and C=O bending, $v_{26}^{\prime }\left(a\right)$ modes.

### 3.6. Potential Energy Curves for C=O Stretching Coordinate and C2-C4-C3 Dihedral Angle

**Figure 5:**
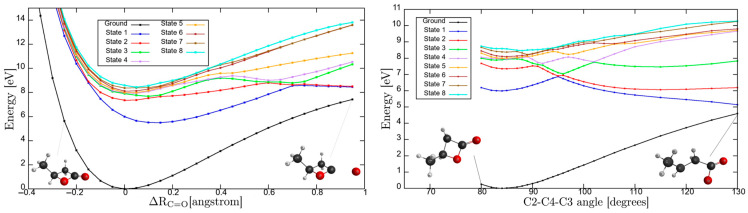
Potential energy curves for the ground and the eight lowest-lying singlet excited states of β-butyrolactone, following the C=O stretching, $v_{22}^{\prime }\left(a\right)$ coordinate (in Å) and C2-C4-C3 dihedral angle (in degrees), while maintaining all other internuclear distances and angles frozen at the ground state equilibrium values. The calculations were performed at the TD-DFT/CAM-B3LYP/aug-cc-pVDZ level of theory in the *C*_1_ symmetry group. See text for details.

The nuclear dynamics governing the intricate molecular landscape is responsible for a relevant instability in β-butyrolactone ring integrity upon electronic excitation. To further our knowledge on the underlying mechanism responsible for ring opening, we have performed calculations at the TD-DFT/CAM-B3LYP/aug-cc-pVDZ level of theory in the *C*_1_ symmetry group. These have allowed the computation of potential energy curves (PECs) for the ground and the eight lowest-lying singlet excited states, following the $v_{22}^{\prime }\left(a\right)$ C=O coordinate and the C2-C4-C3 dihedral angle, while maintaining all other internuclear distances and angles frozen at the ground state equilibrium values, with results shown in Figure 5 (for atom numbering, see Figure S1).

The PECs were computed at the TD-DFT/CAM-B3LYP/aug-cc-pVDZ level, while single-point energies reported in Table 1 were obtained with the larger aug-cc-pVTZ basis. The use of a smaller aug-cc-pVDZ basis for PECs was motivated by its lower computational cost. Table 6 presents the excitation energies calculated with aug-cc-pVDZ, showing good agreement with the aug-cc-pVTZ results for the first eight excited states (Table 1 and Table 6). This consistency indicates that, despite being computed with the smaller basis, the PECs retain a good level of accuracy.

The PECs as a function of $∆R_{C=O}$ can only lead to dissociation if the higher-lying electronic states are attained. The asymptotic limit may be reached if the transition occurs to an electronic state above “state 5”. The calculated electronic transitions up to 6A1 are all mixed valence-Rydberg and Rydberg in character, meaning that all are bound within the reaction coordinate (Table 6). This is consistent with the photoabsorption spectrum in Figure 1, where the bands appear with no background contribution; also relevant is the fine structure involving the C=O stretching, $v_{22}^{\prime }\left(a\right)$ mode (see Table 2). The electronic excitations to “state 6” and/or “state 7” are degenerated at $∆R_{C=O}\approx 0.2\;Å$, and if the reaction coordinate stretches to a value of 0.25 Å, an avoided crossing can be reached. The wavefunction may tunnel through “state 3”, and if it survives long enough that $∆R_{C=O}\approx 0.6\;Å$, the system may dissociate if the adiabatic character is kept. Thus, the nuclear dynamics along these curves either keeps its original Rydberg character or may allow Rydberg-to-valence conversion. It is worth mentioning that none of the calculated electronic states are dissociative in nature. Note that accessing “state 8”, any bond breaking along C=O may be possible through conversion to “state 3”. However, as mentioned in Section 3, and within the Born-Oppenheimer approximation, the electronic excitations in Table S1, although showing an important mixed valence-Rydberg and Rydberg character, less significant $\sigma^{*}$ antibonding MOs can be accessed, thus rendering bond excision. Notwithstanding, one should not discard the importance of strong vibronic coupling within the higher-lying electronic states, which can give rise to enhanced transition probabilities, yielding diffuse spectral features (e.g., Figure 4) with relevant backgrounds.

We now turn our attention to the eight lowest-lying excited states as a function of the C2-C4-C3 angle. Here, avoided crossings are noticeable in the higher excited electronic states, with the inner lying very close to the Franck-Condon region. Electronic excitation from the ground state to “state 1” may lead to ring opening even within the Franck-Condon region. However, that is not supported from the calculation result given the mixed valence-Rydberg character of the state (Table 1 and Table 5). As noted in β-propiolactone [4], such can only be operative through a relevant $\pi_{C=O}^{*}/\sigma_{C=O}^{*}$ coupling or through direct access to a $\sigma_{C-O}^{*}$ antibonding MO. However, accessing “state 2” may lead to C-O dissociation within the adiabatic description above 90°, which becomes even more significant for the higher energy states showing quasi-shallow PECs within the Franck-Condon region. The higher energy states also appear degenerate at the equilibrium geometry, “states 3–6”, whereas any slight change in the C2-C4-C3 angle can lead to bond excision within the adiabatic description. Other degeneracies are also noted, but these require significant change in the C2-C4-C3 angle. We may conclude that not only the electronic but also the molecular structures are relevant, dictating the lack of cohesion within the ring, which will lead to opening upon electronic excitation.

### 3.7. Absolute Photoabsorption Cross Sections and Atmospheric Photolysis

The absolute cross-section values in units of Mb, in the photon energy region 3.8–10.8 eV, are included in Table 1 for the major electronic excitations. However, a literature survey reveals no previous studies of the vacuum ultraviolet photoabsorption of β-butyrolactone to compare with the present work.

The local lifetime of a chemical compound in the terrestrial atmosphere, from sea level (0 km) up to the stratopause (50 km), can be estimated relative to photolysis. This will give an indication as to whether solar exposure is a relevant sink mechanism or if other routes may play a significant role within the atmospheric chemical reactivity of a molecular species. At altitudes below 50 km, the solar radiation reaching the Earth’s atmosphere has wavelengths above 180 nm. The solar actinic flux measurements available from the literature [30] combined with the present absolute cross sections below 6.89 eV (above 180 nm) are used to estimate the photolysis rate (local lifetime) close to 0 km up to the stratopause at 50 km altitude. For further details refer to Limão-Vieira et al. [5]. We are not aware of any photodissociation experiments in the literature to obtain the quantum yield for dissociation, though we shall assume a value following absorption to be unity. The reciprocal of the photolysis rate at a given altitude corresponds to the local photolysis lifetime. Photolysis lifetimes of less than 1 sunlit day were calculated at altitudes above 27 km, showing that β-butyrolactone molecules can be broken up quite efficiently by UV absorption at these altitudes. However, at lower altitudes, lifetimes up to 6 days are expected, with photolysis not playing a relevant sink mechanism.

At 300K, Barnes et al. [3] report gas-phase kinetic studies for β-butyrolactone reactions with ^•^OH radicals. The reaction rate k_OH_ = (0.97 ± 0.26) × 10^−12^ cm^3^ molec^−1^ s^−1^, with the major reactivity occurring through the carbon site C3 adjacent to the heterocyclic oxygen (see Figure 5 and Figure S1). As far as we are aware, no other rate constants are reported to compare with, so currently the major atmospheric sink mechanism of β-butyrolactone may be reactions with the ^•^OH radical [3].

## 4. Materials and Methods

The methodology we have been using to deal with the valence shell electronic state spectroscopy of molecules combines high-resolution vacuum ultraviolet (VUV) photoabsorption experiments with quantum chemical calculations.

The experiments were performed on the AU-UV beam line at the synchrotron light source ASTRID2, Aarhus University, Denmark, in the wavelength range from 115 nm up to 320 nm (10.8–3.9 eV), with a photon resolution better than 0.08 nm [12], corresponding to 1, 3, and 7 meV at the low extreme, the midpoint, and the high extreme of the photon energy range scanned, respectively. The experimental details have been described in detail before [12,31], so only a brief description is given. An absorption gas cell end station with a pathlength of 15.5 cm is used, with the intensity of light passing through it measured either evacuated or filled with a gas sample of β-butyrolactone vapour at room temperature. Two MgF_2_ transmission windows enclosing the cell set the lower wavelength limit of detection (115 nm), while the transmitted light is detected by a photomultiplier tube (PMT). During each absorption scan for a set wavelength region, the absolute pressure of the sample in the absorption cell is measured by a capacitance manometer (Chell CDG100D), giving the molecular number density. This is needed to obtain the absolute photoabsorption cross-section values, *σ*, in units of megabarn (1 Mb ≡ 10^−18^ cm^2^) from the Beer-Lambert attenuation law: $I_{t}=I_{0}e^{\left(-N\sigma l\right)}$, where *I_t_* is the light intensity transmitted through the gas sample, *I_o_* is that through the evacuated cell, *N* the molecular number density of cyclohexane, and *l* the absorption path length. The absolute photoabsorption cross-section values were measured in the pressure range 0.04–1.43 mbar to achieve attenuations of 50% or less and hence avoid saturation effects. The accuracy of the cross-section values is obtained by recording the VUV spectrum in small (5 or 10 nm) sections, allowing an overlap of at least 10 points between the adjoining sections and optimizing the pressure used for the measurement based on the cross-sections of each section, thus allowing us to determine photoabsorption cross-sections to an accuracy of ±5%.

The liquid sample of β-butyrolactone (CAS number: 3068-88-0) used in the VUV photoabsorption measurements was purchased from Aldrich (St. Louis, MO, USA), with a stated purity of 98%. The sample was degassed through repeated freeze-pump-thaw cycles before use.

Ab initio quantum chemical calculations have been performed at the DFT/TD-DFT [32,33] level with a CAM-B3LYP functional [34], and the aug-cc-pVTZ basis set as implemented in the GAMESS-US computational package [35], while those at the EOM-CCSD/aug-cc-pVDZ level have been obtained with Psi4 [36]. The features in the photoabsorption spectrum (Figure 1, Figure 2, Figure 3 and Figure 4) have been assigned with the help of TD-DFT calculations on the vertical excitation energies and oscillator strengths. The major electronic excitations are in Table 1 together with the experimental values and their cross-section values. In Table S1 a complete set of the calculated electronic transitions can be found together with oscillator strengths, while Table S2 contains complementary lowest-lying electronic excitations up to 8.7 eV obtained at the EOM-CCSD/aug-cc-pVDZ level. The calculated harmonic frequencies for the neutral electronic ground state and cation electronic ground state, in Tables S3 and S4 are used to assign the main fine structure in the mixed valence-Rydberg and Rydberg excitations in the photoabsorption spectrum (Figure 1, Figure 2, Figure 3 and Figure 4).

The lowest-lying triplet transition energy was obtained at the TD-DFT/CAM-B3LYP/aug-cc-pVTZ and the EOM-CCSD/aug-cc-pVDZ levels, to be 5.452 and 5.816 eV.

The calculated adiabatic and vertical ionization energies (IE_ad_/IE_v_) were obtained at the DFT/CAM-B3LYP and TD-DFT/CAM-B3LYP levels, with both the aug-cc-pVTZ basis set. The energy values are (IE_1_)_ad_/(IE_1_)_v_ = 10.31/10.56 eV (23a)^−1^, (IE_2_)_v_ = 11.29 eV (22a)^−1^, (IE_3_)_v_ = 12.47 eV (21a)^−1^, (IE_4_)_v_ = 13.19 eV (20a)^−1^, and (IE_5_)_v_ = 13.80 eV (19a)^−1^. Additionally, we have calculated with Psi4 [36] the first vertical ionization energy at the CCSD(T)/aug-cc-pVDZ level to be 10.45 eV. A close comparison between TD-DFT and CCSD(T) lowest-lying vertical ionization energies yields a difference of 1%, thus giving confidence to the calculation methodologies employed.

## 5. Conclusions

We report a novel high-resolution VUV photoabsorption spectrum of β-butyrolactone in the photon energy range from 3.9 to 10.8 eV. The valence-shell electronic state spectroscopy has been investigated together with ab initio calculations at the DFT and TD-DFT levels with the CAM-B3LYP functional and aug-cc-pVTZ basis set. The nature of the electronic excited states has been assigned to mixed valence-Rydberg and Rydberg character based on the visual inspection of the MOs, with the calculations also providing vertical excitation energies and oscillator strengths. The molecular geometries of the neutral ground state and cationic ground state are also reported, giving strong evidence to the fine structure assignments involving the C=O stretching, $v_{7}^{\prime }\left(a\right)$, CH_2_ wagging, $v_{14}^{\prime }\left(a\right)$, C-O stretching, $v_{22}^{\prime }\left(a\right)$ and C=O bending, $v_{26}^{\prime }\left(a\right)$ modes.

The photolysis of β-butyrolactone does not play a significant role in the Earth’s atmosphere below 27 km altitude, whereas gas-phase kinetic studies for reactions with the ^•^OH radical seem to be the major atmospheric sink mechanism. TD-DFT with CAM-B3LYP functional and aug-cc-pVDZ basis set has been used to obtain potential energy curves for the eight lowest-lying singlet excited states along the $v_{22}^{\prime }\left(a\right)$ C=O coordinate and the C2-C4-C3 dihedral angle. An important internal conversion from Rydberg-to-valence character has been reported for the higher-lying electronic states, while upon electronic excitation important carbonyl bond breaking and ring opening prevail. This is an assertion of relevant ring strain instability, a process operative also in β-propiolactone [4].

## Figures and Tables

**Table 1:** The major calculated vertical excitation energies (TD-DFT/CAM-B3LYP/aug-cc-pVTZ) and oscillator strengths of β-butyrolactone, CH_3_CHCH_2_CO_2_, were compared with the present experimental data. Energies in eV. See text for details.

β-Butyrolactone				E (eV) Expt. ^2^	Cross-Section (Mb)
State	E (eV)	*f*_L_	Dominant Excitations ^1^		
$\tilde{X}$ ^1^*A*					
2 ^1^*A*	6.010	0.00047	$\pi^{*}/3p\left(28a\right)\left(18\%\right)+3d\left(29a\right)\left(36\%\right)+3d^{\prime }\left(31a\right)\left(29\%\right)\leftarrow {\bar{n}}_{O}/\sigma_{CC}\left(23a\right)$	6.212	0.34
3 ^1^*A*	7.370	0.02663	$3s\left(27a\right)\left(12\%\right)+3s\left(24a\right)\left(66\%\right)\leftarrow {\bar{n}}_{O}/\sigma_{CC}\left(23a\right)$	7.424	16.63
6 ^1^*A*	7.996	0.06959	$\pi^{*}/3p^{\prime }\left(25a\right)\left(16\%\right)+\pi^{*}/3p\left(28a\right)\left(13\%\right)+3d\left(29a\right)\left(17\%\right)+3d^{\prime }\left(31a\right)\left(17\%\right)\leftarrow n_{O}/\sigma_{CCH_{3}}\left(22a\right)$	7.55(5)	16.21
9 ^1^*A*	8.585	0.03097	$\pi^{*}/3p^{\prime }\left(25a\right)\left(23\%\right)+3p\prime \prime \left(26a\right)\left(26\%\right)+3s\left(27a\right)\left(16\%\right)\leftarrow n_{O}/\sigma_{CCH_{3}}\left(22a\right)$	7.94(5)	13.09
14 ^1^*A*	8.937	0.02543	$3s\left(24a\right)\left(70\%\right)\leftarrow \sigma_{CO}/\sigma_{CC}\left(21a\right)$	8.842	25.58
17 ^1^*A*	9.145	0.03519	$4d\left(32a\right)\left(16\%\right)+4d^{\prime }\left(33a\right)\left(32\%\right)\leftarrow {\bar{n}}_{O}/\sigma_{CC}\left(23a\right)$	9.272	26.03
24 ^1^*A*	9.611	0.02452	$\pi^{*}/3p^{\prime }\left(25a\right)\left(36\%\right)\leftarrow \sigma_{CO}/\sigma_{CC}\left(21a\right)+3d\prime \prime \left(34a\right)\left(18\%\right)\leftarrow {\bar{n}}_{O}/\sigma_{CC}\left(23a\right)$	9.515	32.82
28 ^1^*A*	9.734	0.03017	$3s\left(24a\right)\left(16\%\right)\leftarrow \sigma_{CC}/\sigma_{CO}\left(20a\right)+\left(3d^{\prime }\left(31a\right)\left(20\%\right)+3d\prime \prime \prime \left(33a\right)\left(34\%\right)\right)\leftarrow n_{O}/\sigma_{CCH_{3}}\left(22a\right)$	9.670	35.40
31 ^1^*A*	9.944	0.03858	$nf^{\prime }\left(35a\right)\left(51\%\right)+nf\left(40a\right)\left(13\%\right)\leftarrow {\bar{n}}_{O}/\sigma_{CC}\left(23a\right)$	9.87(1)	41.01
36 ^1^*A*	10.262	0.04253	$3s\left(24a\right)\left(13\%\right)\leftarrow \sigma_{CH}/\pi_{CO}\left(18a\right)+3s\left(24a\right)\left(50\%\right)\leftarrow \sigma_{CCH_{3}}/\pi_{CO}\left(19a\right)$	10.04(7)	47.47
40 ^1^*A*	10.413	0.07819	$3p\prime \prime \left(26a\right)\left(12\%\right)\leftarrow \sigma_{CC}/\sigma_{CO}\left(20a\right)$	10.69(8)	62.27

^1^ it is not possible to give the principal quantum number for the *nf* Rydberg members; ^2^ the last decimal of the energy value is given in brackets for these less resolved features.

**Table 6:** Calculated vertical excitation energies (TD-DFT/CAM-B3LYP/aug-cc-pVDZ) and oscillator strengths (*f*_L_) of β-butyrolactone, CH_3_CHCH_2_CO_2_, for C=O stretching, $v_{22}^{\prime }\left(a\right)$ mode while allowing all atoms to relax. Energies in eV.

State	E (eV)	*f*_L_	Dominant Excitations
2 ^1^*A*	5.991	0.00047	$3d^{\prime }\left(30a\right)\left(36\%\right)+3d\left(29a\right)\left(11\%\right)+\pi^{*}/3p\left(28a\right)\left(35\%\right)\leftarrow {\bar{n}}_{O}/\sigma_{CC}\left(23a\right)$
3 ^1^*A*	7.356	0.02715	$3s\left(27a\right)\left(11\%\right)+3p\prime \prime \left(26a\right)\left(10\%\right)+3s\left(24a\right)\left(69\%\right)\leftarrow {\bar{n}}_{O}/\sigma_{CC}\left(23a\right)$
4 ^1^*A*	7.902	0.00844	$3s\left(24a\right)\left(77\%\right)\leftarrow n_{O}/\sigma_{CCH_{3}}\left(22a\right)$
5 ^1^*A*	7.955	0.02107	$3p\prime \prime \left(26a\right)\left(19\%\right)+3s\left(25a\right)\left(46\%\right)\leftarrow {\bar{n}}_{O}/\sigma_{CC}\left(23a\right)$
6 ^1^*A*	7.993	0.06621	$3d^{\prime }\left(30a\right)\left(15\%\right)+\pi^{*}/3p\left(28a\right)\left(22\%\right)+\pi^{*}/3p^{\prime }\left(25a\right)\left(19\%\right)\leftarrow n_{O}/\sigma_{CCH_{3}}\left(22a\right)$
7 ^1^*A*	8.106	0.00889	$3d^{\prime }\left(30a\right)\left(10\%\right)+3p\prime \prime \left(26a\right)\left(22\%\right)+3\pi^{*}/3p^{\prime }\left(25a\right)\left(28\%\right)+3s\left(24a\right)\left(14\%\right)\leftarrow {\bar{n}}_{O}/\sigma_{CC}\left(23a\right)$
8 ^1^*A*	8.380	0.01630	$\left(3s\left(27a\right)\left(31\%\right)+3p\prime \prime \left(26a\right)\left(30\%\right)\right)\leftarrow {\bar{n}}_{O}/\sigma_{CC}\left(23a\right)+\pi^{*}/3p^{\prime }\left(25a\right)\left(12\%\right)\leftarrow n_{O}/\sigma_{CCH_{3}}\left(22a\right)$
9 ^1^*A*	8.588	0.03526	$3s\left(27a\right)\left(15\%\right)+3p\prime \prime \left(26a\right)\left(28\%\right)+\pi^{*}/3p^{\prime }\left(25a\right)\left(22\%\right)\leftarrow n_{O}/\sigma_{CCH_{3}}\left(22a\right)$

## Data Availability

Data presented in this publication are available upon request to the authors.

## Supplementary Materials

The following supporting information can be downloaded at: https://www.mdpi.com/article/10.3390/molecules30153137/s1, Figure S1: Neutral ground state geometry and electronic configuration of β-butyrolactone obtained at the DFT/CAM-B3LYP/aug-cc-pVTZ level of theory. Bond lengths are in Å and bond angles in (°); Figure S2: Cationic ground state geometry of β-butyrolactone obtained at the DFT/CAM-B3LYP/aug-cc-pVTZ level of theory. Bond lengths are in Å and bond angles in (°); Figure S3: Representation of the molecular orbitals (TD-DFT/CAM-B3LYP/aug-cc-pVTZ) of β-butyrolactone (*C*_1_); Figure S4: Representation of the molecular orbitals (EOM-CCSD/aug-cc-pVDZ) of β-butyrolactone (*C*_1_); Table S1: The calculated vertical excitation energies (TD-DFT/CAM-B3LYP/aug-cc-pVTZ) and oscillator strengths of β-butyrolactone (energies in eV). See text for details; Table S2: The calculated vertical excitation energies (EOM-CCSD/aug-cc-pVDZ) and oscillator strengths of β-butyrolactone, compared with corresponding experimental data (energies in eV). See text for details; Table S3: Harmonic frequencies calculated at the DFT level with the CAM-B3LYP/aug-cc-pVTZ functional and basis set for β-butyrolactone neutral electronic ground state; Table S4: Harmonic frequencies calculated at the DFT level with the CAM-B3LYP/aug-cc-pVTZ functional and basis set for β-butyrolactone cation electronic ground state.
