# Supplementary material for: Ring Opening upon Valence Shell Excitation in β-Butyrolactone: Experimental and Theoretical Methods
Source: Molecules. 2025 Jul 26;30(15):3137. doi: 10.3390/molecules30153137 (PMC12348474; doi:10.3390/molecules30153137)
Supplement: Supplementary file 1 [file molecules-30-03137-s001.zip › molecules-3750135-supplementary.pdf]

Supplementary Material

Ring opening upon valence shell excitation in β-butyrolactone: experimental and theoretical methods

Pedro A. S. Randi [1], Márcio H. F. Bettega [1,*], Nykola C. Jones [2], Søren V. Hoffmann [2], Małgorzata A. Śmiałek [3,*] and Paulo Limão-Vieira [1,4,*]

[1] Departamento de Física, Universidade Federal do Paraná, Caixa Postal 19044, 81531-980 Curitiba, Paraná, Brazil; pasr@fisica.ufpr.br (P.R.); bettega@fisica.ufpr.br (M.H.F.B.)

[2] ISA, Department of Physics and Astronomy, Aarhus University, Ny Munkegade 120, DK-8000, Aarhus C, Denmark; nykj@phys.au.dk (N.C.J.); vronning@phys.au.dk (S.V.H.)

[3] Faculty of Mechanical Engineering and Ship Technology, Institute of Naval Architecture, Gdansk University of Technology, Narutowicza 11/12, 80-233 Gdansk, Poland; smialek@pg.edu.pl (M.A.S.)

[4] Atomic and Molecular Collisions Laboratory, CEFITEC, Department of Physics, NOVA School of Science and Technology, Universidade NOVA de Lisboa, 2829-516 Caparica, Portugal; plimaovieira@fct.unl.pt

* Correspondence: smialek@pg.edu.pl (M.A.S.); bettega@fisica.ufpr.br (M.H.F.B.); plimaovieira@fct.unl.pt (P.L.-V.); Tel.: +58-347-26-14 (M.A.S.); Tel.: +55-41-3361-3002 (M.H.F.B.); +351-21-294-78-59 (P.L.-V.)

Figure captions

Figure S1. Neutral ground-state geometry and electronic configuration of β-buryrolactone, $CH_3CHCH_2CO_2$, obtained at the DFT/CAM-B3LYP/aug-cc-pVTZ level of theory. Bond lengths are in Å and bond angles in (°).

Figure S2. Cationic ground-state geometry of β-butyrolactone, $CH_3CHCH_2CO_2$, obtained at the DFT/CAM-B3LYP/aug-cc-pVTZ level of theory. Bond lengths are in Å and bond angles in (°).

Figure S3. Representation of the molecular orbitals (TD-DFT/CAM-B3LYP/aug-cc-pVTZ) of β-butyrolactone, $CH_3CHCH_2CO_2$, in the C_1 symmetry group.

Figure S4. Representation of the molecular orbitals (EOM-CCSD/aug-cc-pVDZ) of β-butyrolactone, $CH_3CHCH_2CO_2$, in the C_1 symmetry group.

Table caption

Table S1. The calculated vertical excitation energies (TD-DFT/CAM-B3LYP/aug-cc-pVTZ) and oscillator strengths of β-butyrolactone, $CH_3CHCH_2CO_2$, (energies in eV). See text for details.

Table S2. The calculated vertical excitation energies (EOM-CCSD/aug-cc-pVDZ) and oscillator strengths of β-butyrolactone, $CH_3CHCH_2CO_2$, compared with corresponding experimental data (energies in eV). See text for details.

Table S3. Harmonic frequencies calculated at the DFT level with the CAM-B3LYP/aug-cc-pVTZ functional and basis set for β-butyrolactone, $CH_3CHCH_2CO_2$, neutral electronic ground-state.

Table S4. Harmonic frequencies calculated at the DFT level with the CAM-B3LYP/aug-cc-pVTZ functional and basis set for β-butyrolactone, $CH_3CHCH_2CO_2$, cation electronic ground-state.

Figure S1. Neutral ground-state geometry and electronic configuration of β-buryrolactone, $CH_3CHCH_2CO_2$, obtained at the DFT/CAM-B3LYP/aug-cc-pVTZ level of theory. Bond lengths are in Å and bond angles in (°).

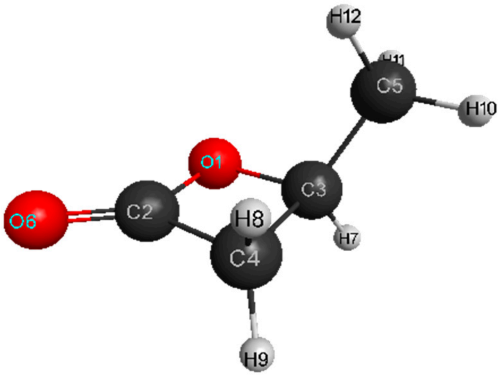

bond length (Å)				angle (°)		
	this work	ref. [1]	ref. [2]		this work	ref. [1]
O1 – C2	1.362	1.388	1.409	O1 – C2 – C3	92.12	92.6
O1 – C3	1.470	1.496	1.455	O1 – C2 – C4	94.31	93.0
C2 – C4	1.516	1.536	1.549	O1 – C3 – C5	111.93	110.5
C3 – C5	1.501	1.510		O1 – C2 – O6	127.83	128.2
C2 – O6	1.184	1.184	1.203	O1 – C3 – H7	108.48	109.7
H7 – C3	1.088	1.075		C2 – C4 – H8	113.79	114.6
C4 – H8	1.087	1.080		H8 – C4 – H9	110.12	
C4 – H9	1.086	1.080		C3 – C5 – H10	109.97	110.1
C5 – H10	1.090	1.084		H10 – C5 - H11	108.28	
C5 – H11	1.089	1.082		H11 – C5 – H12	108.49	
C5 – H12	1.090	1.083				

Electronic configuration of $\tilde{X}\ ^1A$ ground-state:

core orbitals $(1a)^2$ $(2a)^2$ $(3a)^2$ $(4a)^2$ $(5a)^2$ $(6a)^2$;

valence orbitals $(7a)^2$ $(8a)^2$ $(9a)^2$ $(10a)^2$ $(11a)^2$ $(12a)^2$ $(13a)^2$ $(14a)^2$ $(15a)^2$ $(16a)^2$ $(17a)^2$ $(18a)^2$ $(19a)^2$ $(20a)^2$ $(21a)^2$ $(22a)^2$ $(23a)^2$

unoccupied orbitals:

(24a) (25a) (26a) (27a) ...

Figure S2. Cationic ground-state geometry of β-butyrolactone, $CH_3CHCH_2CO_2$, obtained at the DFT/CAM-B3LYP/aug-cc-pVTZ level of theory. Bond lengths are in Å and bond angles in (°).

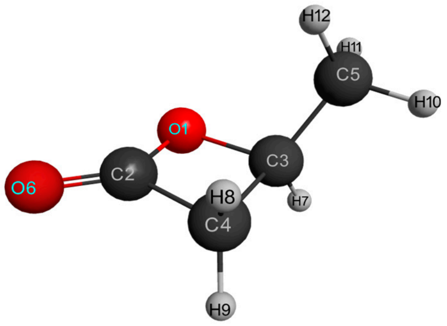

bond length (Å)		angle (°)	
O1 – C2	1.276	O1 – C2 – C3	91.69
O1 – C3	1.544	O1 – C3 – C4	88.13
C3 – C4	1.544	O1 – C3 – C5	111.34
C3 – C5	1.485	O1 – C2 – O6	134.37
C2 – O6	1.212	O1 – C3 – H7	105.50
H3 – C7	1.085	C2 – C4 – H8	110.97
C4 – H8	1.087	H8 – C4 – H9	112.34
C4 – H9	1.086	C3 – C5 – H10	108.45
C5 – H10	1.091	H10 – C5 - H11	107.69
C5 – H11	1.089	H11 – C5 – H12	108.82
C5 – H12	1.089		

Figure S3. Representation of the molecular orbitals (TD-DFT/CAM-B3LYP/aug-cc-pVTZ) of β-butyrolactone, $CH_3CHCH_2CO_2$, in the C_1 symmetry group.

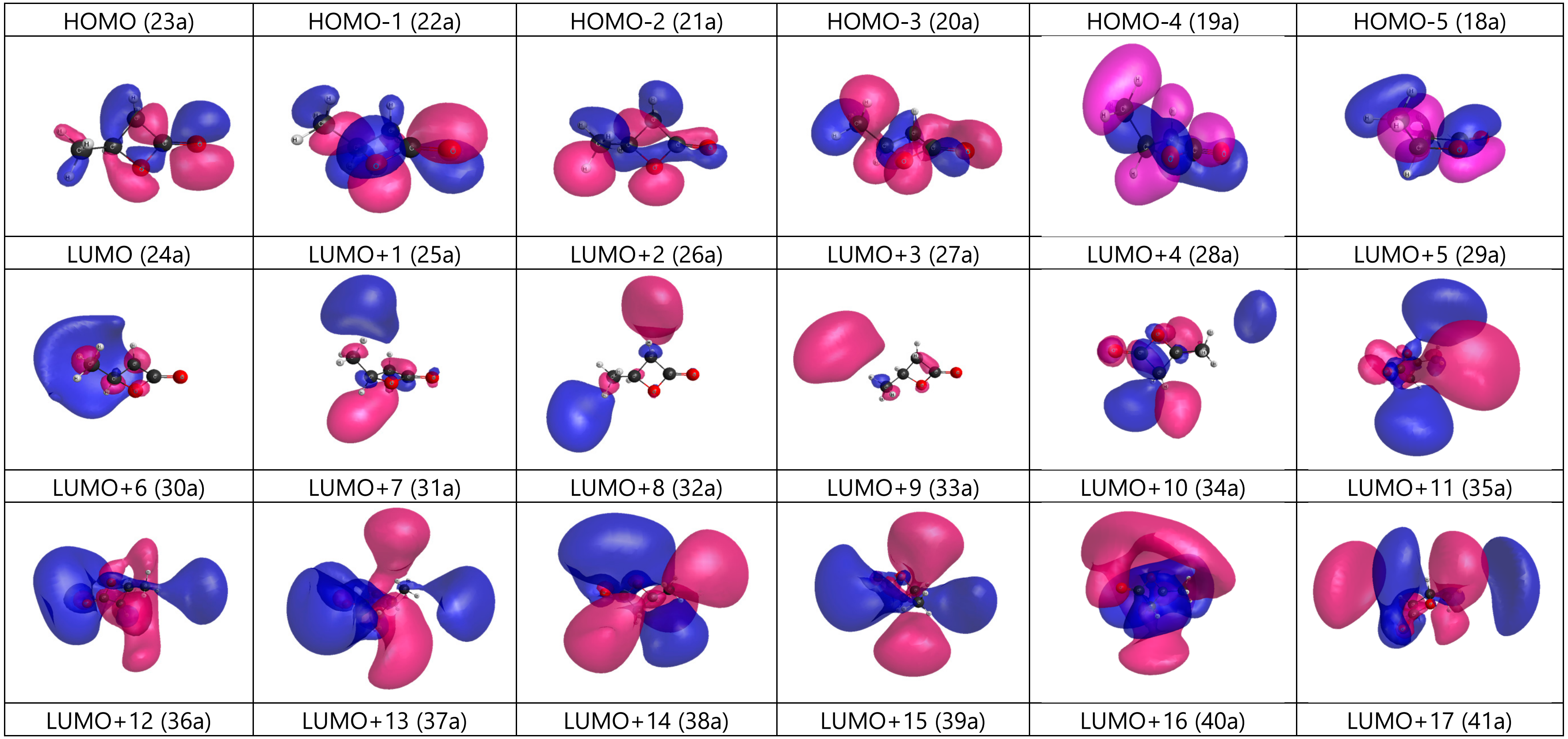

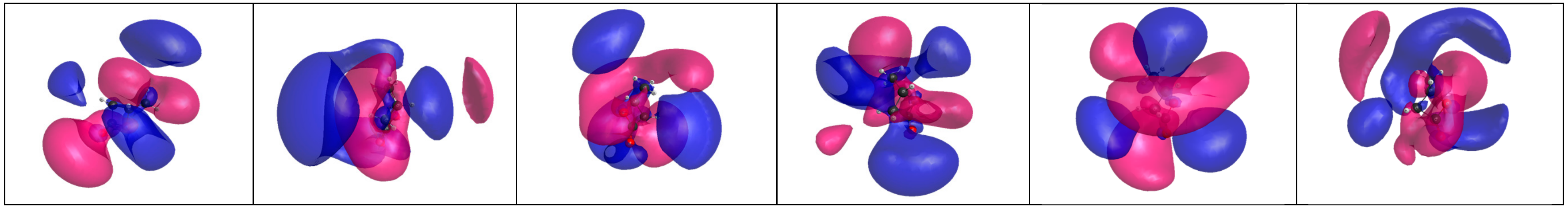

Figure S4. Representation of the molecular orbitals (EOM-CCSD/aug-cc-pVDZ) of β-butyrolactone, $CH_3CHCH_2CO_2$, in the C_1 symmetry group.

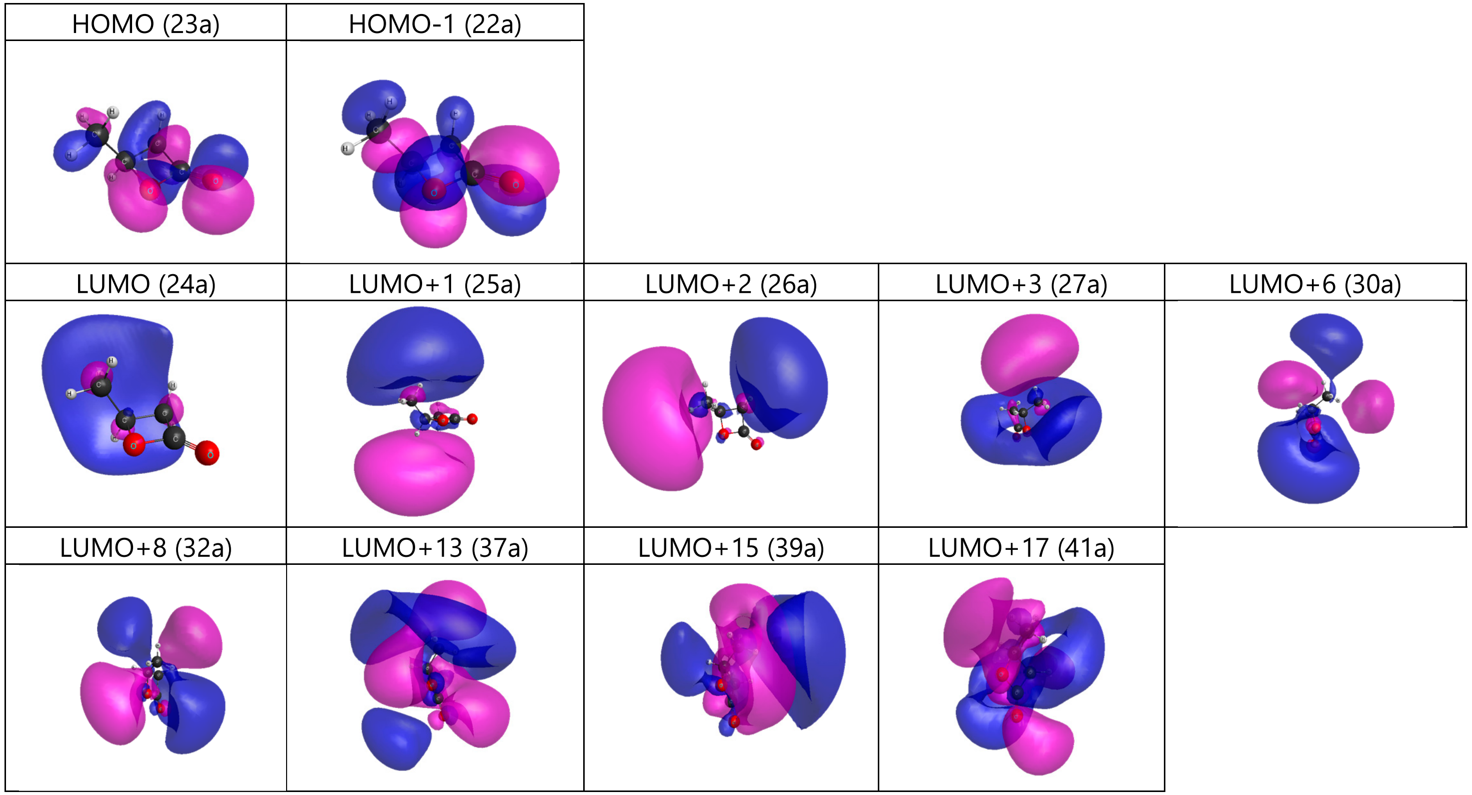

Table S1. The calculated vertical excitation energies (TD-DFT/CAM-B3LYP/aug-cc-pVTZ) and oscillator strengths of β-butyrolactone, $CH_3CHCH_2CO_2$, (energies in eV). See text for details.

β-butyrolactone			
State	E (eV)	f_L	Major excitations
A	6.0103	0.000472	HOMO→L+4 (18%), HOMO→L+6 (36%), HOMO→L+7 (29%)
A	7.3698	0.026630	HOMO→LUMO (66%), HOMO→L+3 (12%)
A	7.9199	0.006512	H-1→LUMO (72%)
A	7.9530	0.016679	HOMO→L+1 (46%), HOMO→L+2 (18%)
A	7.9964	0.069591	H-1→L+1 (16%), H-1→L+4 (13%), H-1→L+6 (17%), H-1→L+7 (17%)
A	8.0968	0.009692	HOMO→LUMO (15%), HOMO→L+1 (29%), HOMO→L+2 (18%), HOMO→L+6 (16%)
A	8.3420	0.017499	HOMO→L+2 (34%), HOMO→L+3 (28%)
A	8.5845	0.030970	H-1→L+1 (23%), H-1→L+2 (26%), H-1→L+3 (16%)
A	8.6159	0.004267	H-2→L+4 (15%), H-2→L+6 (26%), H-2→L+7 (19%)
A	8.6678	0.012226	H-1→L+1 (28%), H-1→L+3 (10%), H-1→L+6 (21%)
A	8.7744	0.004073	H-1→L+2 (41%), H-1→L+3 (26%)
A	8.8146	0.004243	HOMO→L+4 (32%), HOMO→L+5 (15%)
A	8.9365	0.025431	H-2→LUMO (70%)
A	8.9685	0.015670	HOMO→L+4 (26%), HOMO→L+5 (24%), HOMO→L+8 (13%)
A	9.0120	0.019599	HOMO→L+3 (18%), HOMO→L+7 (27%), HOMO→L+8 (15%)
A	9.1448	0.035190	HOMO→L+8 (16%), HOMO→L+9 (32%)

A	9.1897	0.015289	HOMO→L+5 (14%), HOMO→L+8 (22%), HOMO→L+9 (40%)
A	9.3345	0.012165	H-3→L+4 (12%), H-3→L+6 (21%), H-3→L+7 (17%)
A	9.3876	0.002950	H-1→L+4 (25%), H-1→L+5 (23%), H-1→L+6 (13%)
A	9.4168	0.006328	H-1→L+3 (21%), H-1→L+4 (12%), H-1→L+7 (12%), H-1→L+9 (16%)
A	9.5461	0.001290	H-1→L+4 (12%), H-1→L+5 (40%), H-1→L+6 (14%)
A	9.5809	0.008554	H-3→LUMO (10%), H-2→L+1 (12%), HOMO→L+12 (11%)
A	9.6110	0.024521	H-2→L+1 (36%), HOMO→L+10 (18%)
A	9.6339	0.011967	HOMO→L+10 (28%), HOMO→L+12 (20%)
A	9.6527	0.012686	H-3→LUMO (13%), H-2→L+2 (22%), H-1→L+8 (30%)
A	9.6879	0.014606	H-2→L+2 (17%), H-1→L+8 (31%)
A	9.7343	0.030173	H-3→LUMO (16%), H-1→L+7 (20%), H-1→L+9 (34%)
A	9.7697	0.004886	H-3→LUMO (26%), H-2→L+2 (36%)
A	9.8383	0.001979	H-2→L+3 (48%)
A	9.9436	0.038582	HOMO→L+11 (51%), HOMO→L+16 (13%)
A	10.0330	0.004819	H-1→L+9 (20%), H-1→L+12 (24%)
A	10.0486	0.003138	HOMO→L+10 (10%), HOMO→L+13 (52%), HOMO→L+16 (12%)
A	10.1808	0.003390	HOMO→L+14 (57%)
A	10.1891	0.003716	H-1→L+10 (63%)
A	10.2624	0.042525	H-5→LUMO (13%), H-4→LUMO (50%)
A	10.2831	0.041107	H-3→L+1 (54%)
A	10.3463	0.006276	HOMO→L+11 (12%), HOMO→L+16 (29%)
A	10.3757	0.013658	H-2→L+4 (16%)

A	10.4133	0.078194	H-3→L+2 (12%)
A	10.4345	0.013630	H-1→L+11 (43%)
A	10.4650	0.041300	H-5→LUMO (6%), H-3→L+2 (8%), H-2→L+4 (6%), H-1→L+11 (9%), HOMO→L+12 (8%), HOMO→L+15 (8%), HOMO→L+17 (6%)
A	10.4937	0.008452	H-5→LUMO (12%), HOMO→L+12 (10%), HOMO→L+15 (12%)
A	10.5378	0.007379	H-5→LUMO (9%), H-2→L+3 (5%), H-2→L+6 (5%), H-2→L+7 (9%), H-1→L+13 (6%)
A	10.5670	0.057130	H-1→L+13 (19%)
A	10.5968	0.035756	H-5→LUMO (13%), H-3→L+2 (22%)
A	10.6132	0.032339	H-2→L+5 (45%)
A	10.6522	0.015463	H-3→L+3 (17%), H-1→L+13 (28%)
A	10.6689	0.001331	HOMO→L+15 (22%), HOMO→L+17 (31%)
A	10.7303	0.001666	H-6→LUMO (6%), H-3→L+3 (7%), H-4→L+6 (5%), H-2→L+8 (5%), HOMO→L+16 (7%), HOMO→L+20 (7%), HOMO→L+21 (7%)
A	10.8021	0.013139	H-2→L+8 (13%), H-2→L+9 (14%), H-1→L+14 (11%), H-1→L+16 (14%)
A	10.8126	0.006712	H-2→L+8 (11%), H-1→L+14 (23%)
A	10.8423	0.017847	HOMO→L+18 (47%)
A	10.9018	0.017975	H-1→L+16 (10%)
A	10.9247	0.043882	H-6→LUMO (29%), H-2→L+8 (10%)
A	10.9420	0.040065	H-4→L+1 (14%), H-1→L+16 (17%), HOMO→L+21 (12%)
A	10.9655	0.051406	H-4→L+1 (19%), H-1→L+15 (14%)
A	10.9750	0.010228	H-1→L+12 (12%)
A	11.0286	0.004286	H-2→L+9 (15%), H-1→L+12 (10%)
A	11.0675	0.007422	HOMO→L+17 (10%), HOMO→L+19 (12%), HOMO→L+22 (14%)
A	11.0974	0.011070	HOMO→L+17 (12%), HOMO→L+18 (12%), HOMO→L+20 (29%)

Table S2. The calculated vertical excitation energies (EOM-CCSD/aug-cc-pVDZ) and oscillator strengths of β-butyrolactone, $CH_3CHCH_2CO_2$, compared with corresponding experimental data (energies in eV). See text for details.

β-butyrolactone				E (eV) expt.[a]	Cross-section (Mb)
State	E (eV)	f_L	Dominant excitations		
$\tilde{X}$ ^{1}A					
1 ^{1}A	6.144	0.00041680	41a←23a (30%), 39a←23a (3%)	6.212	0.34
2 ^{1}A	7.438	0.03269918	24a←23a (25%), 27a←23a (7%), 26a←23a (3%)	7.424	16.63
3 ^{1}A	7.920	0.00794035	24a←22a (28%), 27a←22a (4%), 37a←22a (3%)	7.55(5)	16.21
4 ^{1}A	8.051	0.03889282	25a←23a (16%), 26a←23a (7%), 41a←22a (5%), 32a←23a (3%)	7.55(5)	16.21
5 ^{1}A	8.076	0.07058933	41a←22a (15%), 25a←22a (4%)	7.95(4)	13.09
6 ^{1}A	8.237	0.01441407	25a←23a (14%), 30a←23a (9%), 24a←23a (6%)		
7 ^{1}A	8.451	0.01448643	26a←23a (19%), 25a←22a (5%), 27a←23a (5%), 30a←23a (3%)		
8 ^{1}A	8.630	0.05149007	27a←22a (11%), 26a←22a (9%), 30a←22a (5%), 25a←22a (5%)	8.842	25.58

[a] the last decimal of the energy value is given in brackets for these less-resolved features;

Table S3. Harmonic frequencies calculated at the DFT level with the CAM-B3LYP/aug-cc-pVTZ functional and basis set for β-butyrolactone, $CH_3CHCH_2CO_2$, neutral electronic ground-state.

$\tilde{X}\ ^1A$					
this work		mode	ref. [3]	ref. [4,5]	assignment
cm^{-1}	eV		cm^{-1}	cm^{-1}	
3168.61	0.393	ν_1(a)	3015 [a]		CH_2 antisymmetric stretching
3135.79	0.389	ν_2(a)	2990		CH_3 antisymmetric stretching (H10 and H12)
3126.69	0.388	ν_3(a)	2980		CH_3 antisymmetric stretching (H11 and H12)
3110.67	0.386	ν_4(a)	2980		CH_2 symmetric stretching
3101.65	0.385	ν_5(a)	2933/2934 [a]		C3–H7 stretching
3056.85	0.379	ν_6(a)	2890		CH_3 symmetric stretching
1938.59	0.240	ν_7(a)	1861		C=O stretching
1503.15	0.186	ν_8(a)	1453/1455 [a]	1441	CH_3 deformation (scissor of H11 and H12)
1493.34	0.185	ν_9(a)	1425	1419	CH_3 deformation (scissor of H10 and H12)
1473.05	0.183	ν_{10}(a)	1391	1387	CH_2 deformation
1433.82	0.178	ν_{11}(a)	1357	1350	CH_3 umbrella
1401.17	0.174	ν_{12}(a)	1288	1284	C3–H bending out-of-plane
1336.41	0.166	ν_{13}(a)	1200	1198	C3–H bending in-plane + C2–O1 stretching
1243.74	0.154	ν_{14}(a)	1183/1184 [a]	1178	CH_2 wagging
1215.76	0.151	ν_{15}(a)	1128/1130 [a]	1118	CH_2 twisting + CH_3 wagging
1162.56	0.144	ν_{16}(a)	1111	1099	ring deformation (C–O)
1131.62	0.140	ν_{17}(a)	1062 [a]	1055	ring deformation + C–CH_3 stretching
1086.84	0.135	ν_{18}(a)	1024	1022	ring deformation C–C
1063.51	0.132	ν_{19}(a)	963		CH_3 wagging + C–O stretching
988.25	0.123	ν_{20}(a)	955	959	ring breathing
923.72	0.115	ν_{21}(a)	901/903 [a]	896	CH_2 rocking + C–H bending out-of-plane
891.57	0.111	ν_{22}(a)	849	836	C–O stretching
836.27	0.104	ν_{23}(a)	816	812	C–CH_2 stretching + C–O stretching
723.51	0.090	ν_{24}(a)	710/713 [a]	711	ring deformation
534.02	0.066	ν_{25}(a)	529		C=O bending (out-of-plane) + CH_2 twisting
518.43	0.064	ν_{26}(a)	515		C=O bending (in-plane)
433.42	0.054	ν_{27}(a)	440		C–CH_3 bending out-of-plane
324.26	0.040	ν_{28}(a)	323		C–CH_3 bending in-plane
237.44	0.029	ν_{29}(a)	223		CH_3 torsion

116.28	0.014	ν_{30}(a)	111		ring puckering

[a] from IR of liquid and/or Raman of liquid [3]

Table S4. Harmonic frequencies calculated at the DFT level with the CAM-B3LYP/aug-cc-pVTZ functional and basis set for β-butyrolactone, $CH_3CHCH_2CO_2$, cation electronic ground-state.

$\tilde{X}\ ^2A$			
this work		mode	assignment
cm^{-1}	eV		
3191.61	0.396	ν_1(a)	CH_2 antisymmetric stretching
3168.48	0.393	ν_2(a)	C3–H7 stretching
3144.83	0.390	ν_3(a)	CH_3 antisymmetric stretching (H10 and H12)
3129.41	0.388	ν_4(a)	CH_3 antisymmetric stretching (H11 and H12)
3115.59	0.386	ν_5(a)	CH_2 symmetric stretching
3058.29	0.379	ν_6(a)	CH_3 symmetric stretching
1594.42	0.198	ν_7(a)	C=O stretching
1490.67	0.185	ν_8(a)	CH_3 deformation (scissor of H11 and H12)
1479.51	0.183	ν_9(a)	CH_3 deformation (scissor of H10 and H12)
1451.82	0.180	ν_{10}(a)	CH_2 deformation
1429.42	0.177	ν_{11}(a)	CH_3 umbrella
1388.18	0.172	ν_{12}(a)	C3–H bending (perpendicular to plane)
1293.36	0.160	ν_{13}(a)	C3–H bending (parallel to plane) + C2–O1 stretching
1231.67	0.153	ν_{14}(a)	CH_2 wagging
1197.64	0.149	ν_{15}(a)	CH_2 twisting + CH_3 wagging
1117.81	0.139	ν_{16}(a)	ring deformation (C–C) + C–CH_3 stretching
1095.81	0.136	ν_{17}(a)	ring deformation + C–CH_3 stretching
1029.95	0.128	ν_{18}(a)	ring deformation C–C
1014.45	0.126	ν_{19}(a)	CH_3 wagging + C–O stretching
914.96	0.113	ν_{20}(a)	CH_2 rocking + C–H bending (perpendicular to plane)
863.14	0.107	ν_{21}(a)	ring breathing
774.88	0.096	ν_{22}(a)	C–O stretching
680.58	0.084	ν_{23}(a)	C–CH_2 stretching + C–O stretching
537.67	0.067	ν_{24}(a)	C=O bending (out-of-plane) + CH_2 twisting
532.46	0.066	ν_{25}(a)	C2–C4 stretching
415.80	0.052	ν_{26}(a)	C–CH_3 bending (perpendicular to plane)
395.54	0.049	ν_{27}(a)	C=O bending (in-plane)
298.88	0.037	ν_{28}(a)	C–CH_3 bending (parallel to plane) + C=O bending
232.05	0.029	ν_{29}(a)	CH_3 torsion
124.45	0.015	ν_{30}(a)	ring puckering

References

1. Gonzalez, E.; Lopez, J.C.; Alonso, J.L. Internal Rotation and Structure of β-Butyrolactone: Microwave and Ab Initio Studies. J. Mol. Structr. 1990, 223, 365–373.
2. Noels, A.F.; Herman, J.J.; Teyssié, P.; André, J.M.; Delhalle, J.; Fripiat, J.G. The Electronic Structure of β-Propiolactone (2-Oxetanone) and Some 3- and 4-Substituted 2-Oxetanones. *J. Mol. Struct. THEOCHEM* **1984**, *109*, 293–303.
3. Durig, J.R.; Morrissey, A.C. Vibrational Spectra and Structure of Small-Ring Compounds. XI. β-Butyrolactone. *J. Mol. Structr.* **1968**, *2*, 377–390.
4. Stephens, P.J.; Devlin, F.J.; Chabalowski, C.F.; Frisch, M.J. Ab Initio Calculation of Vibrational Absorption and Circular Dichroism Spectra Using Density Functional Force Fields. *J. Phys. Chem.* **1994**, *98*, 11623–11627.
5. Stephens, P.J.; Devlin, F.J.; Ashvar, C.S.; Chabalowski, C.F.; Frisch, M.J. Theoretical Calculation of Vibrational Circular Dichroism Spectra. *Faraday Discuss.* **1994**, *99*, 103–119.
